# Deciphering the Role of p53 and TAp73 in Neuroblastoma: From Pathogenesis to Treatment

**DOI:** 10.3390/cancers14246212

**Published:** 2022-12-16

**Authors:** Joana Almeida, Inês Mota, Jan Skoda, Emília Sousa, Honorina Cidade, Lucília Saraiva

**Affiliations:** 1 LAQV/REQUIMTE, Laboratory of Microbiology, Department of Biological Sciences, Faculty of Pharmacy, University of Porto, Rua de Jorge Viterbo Ferreira 228, 4050-313 Porto, Portugal; 2 Department of Experimental Biology, Faculty of Science, Masaryk University, 62500 Brno, Czech Republic; 3 International Clinical Research Center, St. Anne’s University Hospital, 65691 Brno, Czech Republic; 4 Laboratory of Organic and Pharmaceutical Chemistry, Department of Chemical Sciences, Faculty of Pharmacy, University of Porto, Rua de Jorge Viterbo Ferreira 228, 4050-313 Porto, Portugal; 5 Interdisciplinary Centre of Marine and Environmental Research (CIIMAR), Terminal de Cruzeiros do Porto de Leixões, Av. General Norton de Matos s/n, 4450-208 Matosinhos, Portugal

**Keywords:** neuroblastoma, p53 family proteins, N-MYC, miRNAs, targeted anticancer therapy

## Abstract

**Simple Summary:**

Neuroblastoma is the most common extracranial pediatric tumor. Although children with low- and intermediate-risk neuroblastoma, which correspond to approximately half of all newly diagnosed cases, have a good event-free and overall survival, high-risk neuroblastoma can be extremely aggressive and hard-to-treat tumors. In neuroblastoma, p53 and TAp73 act as safeguards against malignant transformation, but they are commonly inhibited by negative regulators, such as MDMs, Itch, and Aurora kinase A. This review focuses on the relevant tumor suppressor role of p53 and TAp73 in neuroblastoma, further addressing their connection with crucial biomarkers of poor prognosis, such as N-MYC, and their great potential as therapeutic targets.

**Abstract:**

Neuroblastoma (NB) is an embryonic cancer that develops from neural crest stem cells, being one of the most common malignancies in children. The clinical manifestation of this disease is highly variable, ranging from spontaneous regression to increased aggressiveness, which makes it a major therapeutic challenge in pediatric oncology. The p53 family proteins p53 and TAp73 play a key role in protecting cells against genomic instability and malignant transformation. However, in NB, their activities are commonly inhibited by interacting proteins such as murine double minute (MDM)2 and MDMX, mutant p53, ΔNp73, Itch, and Aurora kinase A. The interplay between the p53/TAp73 pathway and N-MYC, a known biomarker of poor prognosis and drug resistance in NB, also proves to be decisive in the pathogenesis of this tumor. More recently, a strong crosstalk between microRNAs (miRNAs) and p53/TAp73 has been established, which has been the focused of great attention because of its potential for developing new therapeutic strategies. Collectively, this review provides an updated overview about the critical role of the p53/TAp73 pathway in the pathogenesis of NB, highlighting encouraging clues for the advance of alternative NB targeted therapies.

## 1. Introduction

Neuroblastoma (NB) is a pediatric solid cancer with high prevalence in children younger than 10 years. It is considered one of the most frequent childhood tumors, accounting for 6–10% of all pediatric malignancies [1,2,3]. NB arises anywhere along the developing sympathetic nervous system from neural crest cells or their derivatives, such as Schwann cell precursors [4,5,6]. These cells normally migrate from the dorsal tube and differentiate to tissues or organs of the sympathetic nervous system; when this process fails, NB can be developed. For this reason, this solid tumor tends to appear in regions of the sympathetic nervous system, mainly in the abdomen and adrenal gland [7].

The heterogeneity of NB, which is reflected by several chromosomal aberrations, is considered a hallmark of this disease. This makes its treatment very challenging, especially due to frequent inter- and intra-tumorigenic heterogeneity in patients and the accumulation of gene mutations in recurrent tumor tissues [8]. Targeted therapy is being studied as a promising approach for the treatment of NB, particularly in high-risk patients.

One of the most known genetic factors for the development of high-risk NB is *MYCN* amplification [9,10,11]. Indeed, aberrant overexpression of the *MYCN* oncogene is associated with poor prognosis, tumor aggressiveness, and resistance to chemotherapy [9,10,11]. Another well-known genetic factor in NB is the *anaplastic lymphoma kinase* (*ALK*) mutation or amplification [12]. Most of the otherwise rare familial NB tumors (representing 1% of all NB cases) are associated with *ALK* germline mutations [13,14]. The resulting aberrant activity of ALK contributes to cell growth and survival of cancer cells by induction of pathways such as the phosphoinositide 3-kinase/AKT/mammalian target of rapamycin (PI3K/AKT/mTOR) and RAS/mitogen activated protein kinase (MAPK) [15]. In fact, over 10% of *MYCN*-amplified tumors bear *ALK* mutation [6,10]. The *paired-like homeobox 2B* (*PHOX2B*) is a transcription factor involved in the regulation of differentiation in the sympathetic nervous system [16]. Germline mutations of the *PHOX2B* gene, which result in a loss-of-function protein, predispose to NB development [17,18]. The loss-of-function of the RNA-helicase ATRX by a structural variant is another mechanism involved in the development of NB. These alterations commonly appear in older patients, and they are usually mutually exclusive of *MYCN* amplification [7,10]. Furthermore, in patients with poor outcome and high-risk NB, the *telomerase reverse transcriptase* (*TERT*) is activated by genetic rearrangements [19]. Once activated, telomere lengthening occurs, which might be correlated to the aggressiveness of some types of NB [19]. NB prognosis may also be associated with altered expression of tropomyosin receptor kinase (Trk) proteins. In particular, TrkB is overexpressed in NB cases with amplified *MYCN*, being its activation related to increased proliferation, angiogenesis and chemoresistance of NB cells [20]. Despite this, higher expression of TrkA can also be found in types of NB diagnosed at early ages and without *MYCN* amplification, therefore being associated with better outcomes [10,11]. Some compounds targeting these pathways have already entered clinical trials (Table 1).

Chromosome gain and loss are also related to NB development. In particular, gain of parts of the chromosome 17q and loss of chromosome 1p are associated with *MYCN* amplification in NB, as well as poor prognosis [7]. On the other hand, loss of 11q is inversely correlated with *MYCN* amplification. However, this chromosomal loss is also associated with poor prognosis in NB [21,22].

Loss of heterozygosity (LOH) of 1p36 is common in high-risk NB. Interestingly, the smallest deleted region shared across NB tumors comprises *TP73*, encoding the tumor suppressor protein TAp73 [23]. Indeed, LOH of *TP73* was associated with *MYCN* amplification and subsequently a high-risk NB [24].

The p53 and TAp73 proteins function as molecular hubs of an intricated and robust carcinogenic signaling network, coordinating cell proliferation, death, and differentiation, among many other pivotal cellular processes [25]. This review addresses our current understanding of the p53 and TAp73 pathways in NB, reinforcing their potential as therapeutic targets for the development of new effective drugs against this malignancy.

## 2. The p53 Family Proteins: p53 and TAp73

**Figure 1:**
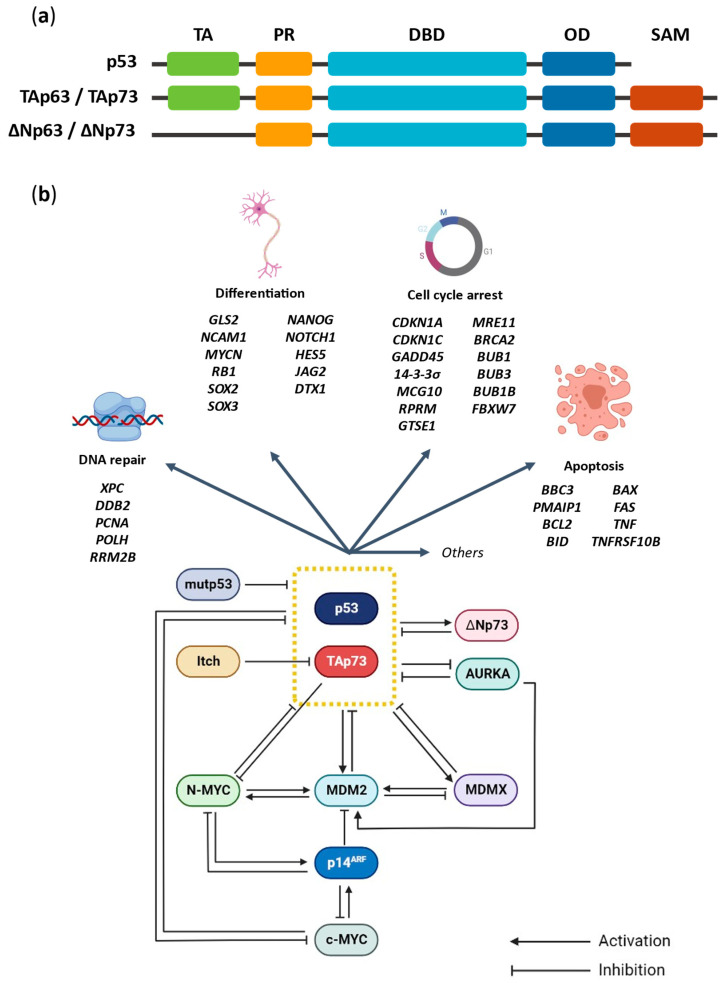
Structure of p53 family proteins: regulation and cellular outcomes of p53 and TAp73 in NB. (**a**) Each protein can be divided into different parts: the *N*-terminal transactivation domain (TA), a proline-rich region (PR), a central DNA-binding domain (DBD), the *C*-terminal oligomerization domain (OD) and the *C*-terminal sterile-α motif (SAM, involved in protein–protein interactions; only present in *TP63* and *TP73*). (**b**) p53 and TAp73 are regulated through interaction with several players, including N-MYC, c-MYC, MDM2, MDMX, AURKA, mutant p53 (mutp53), ITCH and ΔNp73, transcriptionally regulating several downstream targets; the common target genes to p53 and TAp73, in NB, are represented below each cellular outcome.

The p53 family genes *TP53, TP63* and *TP73* (located at chromosome 17p13.1, 3q27–29 and 1p36.2–3, respectively) encode proteins with similar structures (reviewed in [26]) (Figure 1a). Yet, all members have isoforms with different activities. The full-length isoforms TAp63 and TAp73, containing the transactivation (TA) domain (Figure 1a), function as transcription factors with similar tumor suppressor proprieties to p53. In fact, TAp63 and TAp73 DNA-binding domain (DBD) share a high degree of sequence homology (around 60–63%) with p53, which suggests that they may bind to the same DNA sequence and transactivate the same set of target genes [27] (Figure 1b). Despite this, each one has specific functions; for instance, TAp63 is involved in epithelia development and morphogenesis, while TAp73 has an important role in neurogenesis and neural differentiation [28,29]. On the other hand, the *N*-terminally truncated isoforms (∆Np63 and ∆Np73), lacking part or the whole TA domain (Figure 1a), act as dominant negative regulators of p53, TAp63 and TAp73 [30,31,32].

The basal levels of p53 family proteins in cellular homeostasis are usually low; however, their stability and transcriptional activity increases upon stress stimuli. Phosphorylation of the TA domain of p53 and TAp73 by ataxia telangiectasia mutated (ATM) and DNA-dependent protein kinase (DNA-PK), activates these proteins, therefore regulating the expression and activity of their downstream targets involved in distinct cellular responses to prevent the growth or migration of malignant cells [33,34,35] (Figure 1b).

The role of TAp73 in neuronal differentiation further supports the implication of the loss of TAp73 activity in NB development [36]. In fact, it has been shown that TAp73 expression is increased during the differentiation process induced by retinoic acid, which is currently used in NB maintenance therapy [37], while TAp73 depletion inhibits differentiation [36]. This may indicate that the TAp73 activity is functionally associated with the growth inhibition observed during NB differentiation.

Although it has been shown that p53 is not functional in many NB cases, this protein is rarely mutated in NB [38]. Likewise in other cancers, TAp73 is also rarely mutated in NB [39], although some primary NBs show amino acid substitutions (P405R and P425L) in *TP73* [40]. Still, LOH of TAp73 is more common in NB [24]. These data have suggested that the activity of p53 and TAp73 is mainly repressed in NB by other mechanisms, particularly by interaction with negative regulators (Figure 1b).

### 2.1. p53 and TAp73 Interaction with MDM2 and MDMX

Several studies have demonstrated that murine double minute (MDM)2 has a pathogenic role in NB, and that targeting this regulator could be an interesting therapeutic approach. In addition, MDM2 overexpression in NB is more prevalent in relapsed cases rather than in primary NB, indicating a correlation between MDM2 overexpression and poor prognosis in NB [41].

NB commonly harbors a wild-type (wt)p53 form, which is inactivated by interaction with inhibitory proteins such as MDM2, and its homologue MDMX (reviewed in [27,42]) (Figure 1b). In fact, in many types of cancer, including NB [43], MDM2 and MDMX are overexpressed or amplified, acting as oncogenes, and leading to therapeutic resistance and metastasis (reviewed in [44,45,46,47]). The inhibition of TAp73 by these endogenous regulators has also been related to NB, especially in high-risk and relapsed cases [45].

By binding to p53 and TAp73, MDM2 and MDMX can inhibit their transcriptional activity. MDM2 can also bind to p53 and act as an E3 ubiquitin ligase, targeting p53 for proteasomal degradation. In fact, MDM2 mediates the nuclear export of p53 to the cytosol, where it can be mono- or polyubiquitinylated. It should be noted that MDM2 is transcriptionally induced by p53, forming a feedback loop [48,49]. Interestingly, conversely to previous data, a recent study demonstrated that TAp73 may also be degraded in vitro and in vivo, through polyubiquitination by MDM2, being Lys11, Lys29 and Lys63 residues the main targets of MDM2 for TAp73 degradation [50]. Although MDMX does not have ubiquitin ligase activity, it can form a heterodimer with MDM2, promoting this activity by MDM2 and being itself inhibited by MDM2 (reviewed in [42,47]) (Figure 1b). Unlike p53, TAp73 does not undergo MDM2-mediated nuclear export. Instead, TAp73 accumulates in the nucleus of MDM2-expressing cells as aggregates [51], suggesting a structural variation between p53 and TAp73 that differentiates this subcellular distribution. Importantly, MDM2 overexpression has been shown to promote drug resistance in wtp53-proficient NB cells via inactivating p53/TAp73, which results in the transcriptional downregulation of the pro-apoptotic protein NOXA [52].

**Figure 2:**
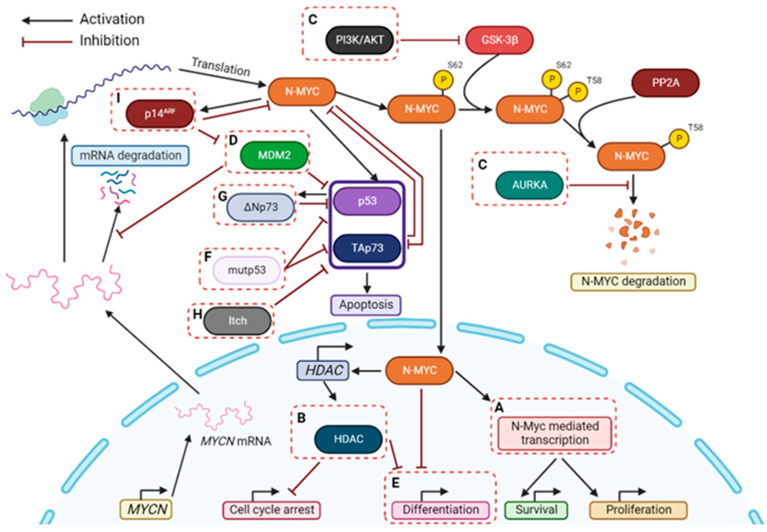
Therapeutic strategies for targeting NB, particularly with *MYCN* amplification. Some possible strategies to treat *MYCN*-amplified NB patients are highlighted in dashed boxes and may include: (**A**) Inhibition of N-MYC-dependent transcription with BET-bromodomain inhibitors; (**B**) Inhibition of HDACs; (**C**) Inhibition of proteins involved in stabilizing N-MYC; (**D**) Suppression of MDM2 (which stabilizes *MYCN* mRNA and disrupts p53-mediated apoptosis); (**E**) Induction of differentiation; (**F**) Destabilization of mutp53-TAp73 interaction; (**G**) Inhibition of ΔNp73; (**H**) Inhibition of Itch; (**I**) Activation of p14ARF. P: Phosphorylation.

Preclinical studies have evidenced that NB cells are very sensitive to inhibitors of MDM2, such as nutlin-3a [53,54,55] (Figure 2). Despite this, the number of MDM2 inhibitors under clinical trials for NB treatment is very limited, only comprising one ongoing study with Idasanutlin (also called RG7388) (Table 1). In fact, some concerns related to the use of these compounds, particularly the development of drug resistance and tumor relapse, have been reported in several studies and are closely associated with the appearance of mutated p53 forms upon long-term therapeutic exposures [45].

An important regulator of the p53-MDM2 network is the tumor suppressor p14^ARF^. By forming a complex with MDM2, p14^ARF^ inhibits MDM2-dependent p53 degradation and maintains p53 in an active state [56] (Figure 1b). In fact, deletion or downregulation of the *INK4a-ARF* gene (encoding p14^ARF^) have represented a major mechanism of p53 inactivation in NB [57]. Consistently, the loss of p14^ARF^ has been observed in relapse NB tumors [58]. Interestingly, p14^ARF^ can also exhibit p53-independent tumor suppressor activity by directly binding and inhibiting c-MYC and N-MYC [52] (Figure 1b). Stimulation of p14^ARF^ would therefore represent an additional therapeutic strategy against NB (Figure 2).

### 2.2. p53 and TAp73 Interaction with Mutant p53

Most p53 mutations occur in the p53 DBD via single amino acid substitutions (missense mutations), which are translated into loss-of-function of the tumor suppressor capacity, or in some cases into oncogenic gain-of-function (GOF) (reviewed in [59]). Mutations in this region also lead to cellular accumulation of mutant (mut)p53, since MDM2 cannot recognize mutp53 and initiate ubiquitin-mediated degradation (reviewed in [59]). Cancers with mutp53 are also associated with increased proliferation, metastasis, angiogenesis, genomic instability, and resistance to therapy [59,60].

Mutp53 is an inhibitor of various transcription factors, including wtp53 [61,62] and TAp73 [63,64] (Figure 1b). It is thought that GOF activity of mutp53 depends on these interactions to mediate the transcription of several genes [65]. Besides affecting transcription factors, mutp53 can also promote the stability of microRNAs (miRNAs) involved in tumor progression and dissemination [66,67,68] (see Section 4).

The TAp73 inhibition by mutp53 is highly related to high-risk and relapsed cancers [69]. In fact, the binding of mutp53 to TAp73 DBD results in the inhibition of TAp73-dependent transactivation and apoptosis [63], and promotion of drug resistance [70,71].

Consistently, even though p53 mutations are rare at diagnosis in NB (<2% in primary tumors and around 15% in relapsed ones) [72,73], various reports have shown that they are associated with the development of drug resistance [74,75]. In line with this, most of the reported mutp53-related NB clinical cases are associated with relapse and poor outcome [72,76,77]. It has also been shown that cytotoxic therapy for NB, including doxorubicin, cisplatin, and vincristine, can induce p53 mutations. In fact, NB cells derived from the same patient before (SK-N-BE(1)) and after (SK-N-BE(2)) chemotherapy, showed alterations in the p53 status (from wt to mut) and therapeutic response (from chemosensitive to chemoresistant) [78]. Indeed, acquired drug resistance has been a major barrier to the successful treatment of many cancers, including NB [79].

Hence, disruption of the mutp53 interaction with transcription factors such as p53 and TAp73 reveals great therapeutic potential against mutp53-profecient NB tumors (Figure 2). In fact, the treatment of cancer cells harboring mutp53 with RETRA, a destabilizer of the mutp53-TAp73 interaction, significantly increased TAp73 expression levels and subsequent transcriptional activity [80,81]. More recently, the xanthone derivative 3,4-dimethoxy-9-oxo-9*H*-xanthene-1-carbaldehyde (LEM2) was uncovered as a potent anticancer agent against patient-derived NB cells [82]. LEM2 inhibited both TAp73-mutp53 and TAp73-MDM2 interactions, resulting in TAp73 activation and induction of cell cycle arrest and apoptosis in NB cells. In these cells, LEM2 also showed promising synergistic effects with chemotherapeutics such as doxorubicin and cisplatin [82].

### 2.3. p53 and TAp73 Interaction with ΔNp73

As previously mentioned, ∆Np73 acts as an oncogene that is associated with cancer development, metastasis, and drug resistance [30,83]. In fact, in 2002, Casciano et al., showed that expression of ∆Np73 was related to decreased apoptosis in vivo, being a robust predictor of unfavorable outcome, regardless of age, primary tumor site, stage, chromosome 1p deletion, and amplification of *MYCN* in NB [84]. In NB, ∆Np73 is considered a poor prognosis marker, namely due to its interaction with p53 and TAp73 and subsequent inhibition of their transcriptional activity (reviewed in [85,86]).

In fact, the effect of TAp73 in NB is thought to depend on the ratio between TAp73 and ΔNp73 isoforms. Several mechanisms responsible for deregulating this ratio have been described in NB, including hyper- or hypomethylation, which are crucial events in cell transformation [87]. Since some human malignancies, such as non-Hodgkin lymphoma, display *TP73* silencing due to promoter methylation [88], it was suggested that this type of modification could also account for the decreased levels of TAp73 in NB. However, this idea gained less support based on the impossibility of establishing a correlation between the *TP73* promoter methylation status and the TAp73 expression in NB [89].

Unlike TAp73, ΔNp73 has been reported to be overexpressed in primary NB [84]. These increased ΔNp73 levels might be responsible for the inhibition of the pro-apoptotic activity of p53 [90] and may even block the TAp73 activity, allowing neural cells to escape from the TAp73-mediated differentiation process [36]. ΔNp73 is also capable of inhibiting the activation of ATM and p53, making NB more resistant to chemotherapeutics [91]. From a mechanistic point of view, this overexpression of ΔNp73 might be related to epigenetic modifications, such as hypomethylation of the internal P2 promoter, which controls the transcription of this isoform and has already been observed in NB cells and primary tumors [89,92].

It has become evident that ∆Np73 has high clinical significance as a marker for NB severity [85]. ∆Np73 is not just a relative of p53, but has created its own identity, also becoming an encouraging target for NB therapy (Figure 2).

### 2.4. Proteasomal-Dependent Degradation of TAp73 by Itch

The E3 ubiquitin ligases (E3s) have proven to play a fundamental role in the regulation of cell proliferation, differentiation, and apoptosis. Consistently, genetic alterations and dysfunctions in the E3s activity have been deeply related to tumor progression [93,94]. The HECT-type E3 ubiquitin ligase Itch has been reported to regulate apoptosis, cell growth and inflammation pathways, and some studies have even shown that its dysregulated expression interfered with the apoptotic response induced by conventional chemotherapy [93,94,95]. In fact, the depletion of Itch by siRNA has sensitized lung cancer cells to anti-proliferative effects of gemcitabine [96]. Similarly, RNA interference-mediated downregulation of Itch significantly enhanced suppression of pancreatic cancer growth by gemcitabine in vivo [97]. This was further demonstrated in NB cells by Meng et al., who used in vivo nano-delivery of the Itch *siRNA* in NB xenograft mouse models to sensitize tumor cells to radiotherapy [98]. Itch is responsible for the regulation of the proteasomal-dependent degradation of a group of target proteins, including TAp73 [99] (Figure 1b). Interestingly, from the several E3s that control TAp73 protein levels [100,101,102], Itch is one of the most characterized. Specifically, it stimulates the proteasome-dependent degradation of TAp73 in unstressed cells, keeping its expression levels low in normal conditions [103]. As shown in many cancer cell lines, in response to chemotherapeutic drugs, the induction of TAp73 activity seems to be, at least in part, accomplished through downregulation of Itch [99].

Since most NB cell lines express Itch, it may be possible that TAp73 levels are negatively controlled by an Itch-dependent mechanism, which could explain the chemoresistance in NB [9]. As such, targeting Itch could represent a strategy of TAp73 stabilization, thus enhancing pro-apoptotic activity of TAp73 and even sensitizing NB cells to commonly used chemotherapeutic drugs. In 2014, Rossi et al. identified desmethylclomipramine, the active metabolite of clomipramine, as an inhibitor of Itch autoubiquitylation activity and Itch-dependent ubiquitylation of TAp73 [104]. Clomipramine is an FDA-approved drug used in the treatment of obsessive-compulsive disorders [105]. Interestingly, this drug also increases the cytotoxic activity of conventional chemotherapeutics in cancer cell lines and cancer stem cells [96,104]. Although it is still unclear if this effect is completely dependent on Itch inhibition, these data suggest that targeting Itch could represent a novel therapeutic approach for NB treatment (Figure 2).

### 2.5. p53 and TAp73 Interaction with AURKA

Aurora kinases are a family of serine/threonine protein kinases that have a crucial role in cellular division. These kinases are essential to ensure the correct replication of the genetic information, as well as for the maintenance of genomic and chromosomal integrity during cell division [106]. The aurora A (AURKA) is the most studied member of Aurora kinase family, mainly due to its central role in mitotic regulation and high expression levels in many types of cancers, including NB [107]. Most of the AURKA proteins are activated in late G2 phase, and their activity is maintained until the end of mitosis [108]. In mitosis, AURKA is mainly involved in centrosome maturation, mitotic entry regulation, and spindle assembly. Once mitosis is completed, most AURKA proteins are degraded, and only a small amount is detected in G1 phase [109].

AURKA is known to acquire gain-of-function alterations, mainly due to amplification, overexpression of its gene and p53 loss-of-function. These events have been associated with several cellular phenotypes, such as centrosome amplification, override of spindle assembly, and DNA damage checkpoint response and aneuploidy [110]. The induction of these phenotypes suggests that AURKA and p53 are involved in overlapping signaling pathways that are responsible for the regulation of these aberrant cellular outcomes. This was first proven in 2002 by Marumoto et al., who demonstrated that p53 was able to suppress the oncogenic effects of AURKA through physiological interaction, in a transactivation-independent manner [111] (Figure 1b). Additionally, p53 has been shown to downregulate AURKA expression, as well as its kinase activity and stability, by binding to AURKA promoter or through activation of p53 target genes, such as *CDKN1A* (encoding p21), *GADD45A* and *FBXW7α*. The induction of p21 inhibits Cdk kinase activity, which leads to the maintenance of RB1 in a hypophosphorylated state in a complex with E2F3. This impairs the *AURKA* gene expression [110]. On the other hand, GADD45 inhibits AURKA kinase activity through direct interaction, preventing cells from centrosome amplification and aborted cytokinesis [112]. These results suggest that the inhibition of AURKA by p53 is important for the maintenance of centrosome number and chromosomal and genomic stability. Regarding the tumor suppressor protein FBXW7α, this p53-dependent protein is a component of the SCF-like ubiquitin ligase complex that targets both AURKA and AURKB for proteasomal degradation [113,114,115]. FBXW7α is frequently downregulated or mutated in tumors. It cooperates with PTEN in the regulation of AURKA degradation via the PI3K/AKT/GSK3β pathway, mainly participating in the degradation of active AURKA proteins [114,116]. The dysfunction of the p53-FBXW7α axis is frequently observed in human tumors, and it has been proven that this deregulation can mediate AURKA-induced centrosome amplification, leading to aneuploidy [114,117]. It should also be mentioned that AURKA is able to stabilize N-MYC by interfering with its FBXW7α-mediated degradation, as demonstrated by a synthetic lethal screening of proteins interacting with N-MYC [118]. Even though it was reported that this interaction is independent of AURKA kinase activity, a study by Brockmann et al. demonstrated that inhibitors of AURKA kinase activity could also disrupt the interaction between AURKA and FBXW7α, leading to N-MYC destabilization and tumor regression in a mouse model of N-MYC-driven NB xenograft [119].

Some studies have reported that Aurora kinases negatively regulate p53 through phosphorylation-mediated posttranslational modification of either p53 itself or of interactor proteins that bind to p53, which may result in failure of the DNA damage checkpoint, as well as lack of response to cell death induction in AURKA overexpressing cells [110]. In fact, AURKA phosphorylates p53 at serine 315, which facilitates MDM2-mediated ubiquitination and degradation of p53 [120]. Furthermore, AURKA phosphorylation of p53 at serine 215 inhibits p53 DNA-binding and subsequent transactivation activity [121]. The role of AURKA in TAp73 regulation was first demonstrated, in 2008, by Dar et al., who showed that the treatment with the AURKA inhibitor MLN8054 or knockdown of AURKA, in p53-deficient cells, induced TAp73-mediated apoptosis [122]. Later, in 2012, AURKA was discovered to directly interact with and phosphorylate TAp73 DBD at serine 235, which resulted in the loss of DNA-binding ability and transactivation activity of TAp73 [123]. These events were observed in cells becoming resistant to DNA damage-induced cell death [123].

It has been demonstrated that spindle assembly checkpoint (SAC) override is associated with aberrant AURKA expression, regardless of p53 status in cells [110]. As a result, it is currently unknown if p53 plays a role in AURKA signaling for SAC override. There is some evidence suggesting that p53 is also involved in mitotic cell death and postmitotic checkpoint following aberrant mitosis or spindle damage by interacting with SAC proteins, rather than activating SAC [124,125,126,127]. However, the role of TAp73 interaction with AURKA in SAC is better understood and defined. Some in vitro studies describe a role for TAp73 in G2-M transition, mitotic exit, and mitotic cell death [124,125,126,127], while an in vivo study in transgenic mice lacking TAp73 has suggested that the frequent occurrence of aberrant spindle structure is associated with aneuploidy and chromosome instability [128]. Additionally, biochemical studies have shown that TAp73 interacted with the SAC proteins BUB1, BUB3 and BUBR1, which are crucial for BUB1 and BUBR1 localization at kinetochores and BUBR1 kinase activity [128,129]. These data indicated that TAp73 was directly involved in regulating SAC signaling to maintain chromosomal stability. These results have suggested that AURKA-TAp73 interaction was crucial for a critical step in the SAC inactivation pathway. However, unlike its effect on the MAD2-CDC20 interaction, phosphorylation of TAp73 did not affect the interaction of BUBR1 with CDC20 and its kinetochore localization, which indicated that TAp73 had a role in a distinct pathway to control SAC activation [129].

Cancer cells with ectopic expression of ΔNp73 show abnormal mitotic progression, followed by multipolar spindle and cytokinesis failure, which results in multinucleated cells [110]. However, ΔNp73 seems not to affect either SAC activation in the presence of spindle poison, or interaction with BUBR1 [128,130], suggesting a contribution of ΔNp73 to bypassing SAC. Interestingly, AURKA also interacts with and phosphorylates ΔNp73, but its phosphorylation site seems to be different from TAp73 and remains to be mapped [123]. The role played by this interaction has not yet been elucidated.

In 2021, Yi et al. showed how inhibitors of AURKA, such as alisertib, were highly synergistic with BET bromodomain inhibitors, in NB cells [131]. Consistently, they observed a decreased *MYCN* mRNA levels due to BET bromodomain inhibitors, which downregulate the transcription of *MYCN*, and increased degradation of N-MYC through AURKA inhibitors. They further evidenced the induction of apoptosis and cell cycle arrest in response to this combination, mainly in a context of a functional *TP53*. Interestingly, the results indicated that *TP53* status may be predictive of therapeutic response to AURKAi, in NB cells, since *TP53* loss conferred resistance to alisertib monotherapy. These data therefore reinforce the beneficial effect that activators of the p53 pathway may have in combination with AURKAi. More recently, a study from Nguyen et al. [132] found that selinexor, an inhibitor of the nuclear export protein XPO1, induced p53 phosphorylation at serine 315, an initiating step for p53 degradation undertaken by AURKA, as previously referred. By using alisertib, p53-mediated cell death was enhanced in NB xenograft mouse models.

## 3. The N-MYC and p53/TAp73 Interplay

N-MYC is a protein encoded by the *MYCN* gene, which was first described in 1983 as an oncogene located on chromosome 2p24 [133,134,135]. It belongs to the MYC protein family, together with c-MYC and L-MYC [136]. The dysregulation of MYC oncoproteins is an important aspect of cancer pathogenesis since it can lead to genome instability and initiate malignant transformation [137]. On the other hand, downregulation of MYC proteins results in the induction of transient [138] or, in some cases, sustained [139] loss of neoplastic phenotype. It has been reported that N-MYC and c-MYC have complementary expression, being key factors for the maintenance of pluripotency of tumor cells [140].

In 1993, a study conducted in mouse myeloid leukemia cells that carry a temperature-sensitive p53 protein demonstrated that p53 decreased the c-MYC mRNA levels [141]. Later, it was demonstrated that p53 repressed c-MYC transcription through histone deacetylation [142]. More recently, it was reported that c-MYC inactivates p53 through the c-MYC-Inducible Long noncoding RNA Inactivating P53 (MILIP). MILIP promotes p53 polyubiquitination and turnover by reducing p53 SUMOylation through suppression of tripartite-motif family-like 2 (TRIML2), which is upregulated in diverse cancer types, and supports cell survival and proliferation [143]. In 2002, Watanabe et al. verified that c-MYC also bound to TAp73 and inhibited the TAp73-dependent transactivation. In that study, it was further demonstrated that overexpression of MM1 (a binding partner of c-MYC) stimulated TAp73 transcriptional activity and growth arrest by antagonizing the inhibitory effect of c-MYC on TAp73 [144]. Interestingly, c-MYC has been previously reported to sensitize cells to apoptosis via induction of p14ARF-mediated upregulation of p53 expression, stability, and activity through inhibition of the MDM2-p53 axis [145,146] (Figure 1b). Despite all these findings, most of the studies in NB have been focused on the role of N-MYC, instead of c-Myc, in the regulation of p53 and TAp73, as *MYCN* amplification is a gold standard marker routinely used in clinic for risk assessment of NB. However, we might speculate that NB overexpressing c-MYC might show similar deregulations in the p53/TAp73 pathways, which deserves further investigation.

N-MYC is a transcription factor with a basic helix-loop-helix motif, which forms a complex with the helix-loop-helix leucine zipper protein MAX, a MYC-associated factor, and binds to E-boxes around target genes. It directly regulates the expression of genes responsible for the maintenance of pluripotency, therefore contributing to cell proliferation and cell cycle progression [147]. The expression of N-MYC is crucial for normal neural development. However, while it is expressed during neural crest cell development, the levels of N-MYC are drastically reduced in differentiated adult neural tissue [148]. A study conducted by Knoepfler et al. in mice demonstrated that N-MYC is essential for maintenance and proliferation of neural precursor cells [149]. They further showed that loss of N-MYC disrupted neuronal differentiation, as evidenced by ectopic staining of the neuron specific marker βTUBIII in the cerebrum [149], and led to premature (increased) differentiation, resulting in reduced size of the organs and other defects. On the other hand, tumors with N-MYC overexpression are composed predominantly of highly proliferative neuronal progenitor cells, which suggests that N-MYC promotes proliferation and prevents differentiation of these progenitor cells, resulting in tumor formation in sympathetic nervous system, as demonstrated in tyrosine hydroxylase (TH)-MYCN transgenic mice (N-MYC regulated by TH promoter) [150]. N-MYC accumulation is associated with an accelerated rate of translation that overcomes a H-Ras(G12V)-mediated destabilization of N-MYC [151].

In fact, several clinical observations have evidenced that *MYCN* amplification is the starting event of high-risk NB [152,153,154,155,156]. *MYCN* amplification is frequently present at diagnosis, and it is either subclonal or acquired during disease progression [152,153,154,155,156]. Moreover, the transgenic expression of *MYCN* in migrating neuroectodermal cells of the neural crest using a rat TH promoter triggered NB development in mice [157]. Another study confirmed the TH-MYCN mice using cre-mediated recombination [158]. In that model, iCre is only expressed in dopamine β-hydroxylase-proficient cells (marker of sympathetic neuron differentiation). This triggered recombination in the transgenic locus and expression of N-MYC, which recapitulated NB development in sympathetic ganglia [158]. *MYCN* overexpression was also induced in primary neural crest cells derived from an embryonic neurotube explant [159]. These transduced cells were subcutaneously introduced back to mice, which led to the formation of tumors that phenotypic and molecularly resembled human *MYCN* amplified NB [159]. Ectopic expression of *MYCN* in the neural crest of zebrafish also induced NB, which substantiated that the potential of *MYCN* to induce this type of cancer is conserved among species [160]. Collectively, all these studies have corroborated that an increased expression of N-MYC is a driving factor of NB.

In 2016, Powers et al. suggested that the *MYCN* mRNA might also have an oncogenic role in NB that is independent of N-MYC protein, acting as a competing endogenous RNA (ceRNA). This concept emerged from the studies of miRNA let-7, which targets *MYCN* mRNA for degradation [161]. Expression of LIN28B, which is a RNA-binding protein and an inhibitor of let-7 [162], was shown to maintain high levels of *MYCN* mRNA in *MYCN*-amplified NB cells [163]. The transgenic expression of LIN28B in mouse sympathetic adrenergic lineage, using the *Dbh* promoter [158,164], induced the development of NB tumors that were characterized by low let-7 miRNA levels and high expression of N-MYC [165]. In line with these observations, increased expression of LIN28B also induced tumors in mouse models, including liver [166], colon [166] and Wilms [167] tumors.

The *MYCN* amplification plays a crucial role in the p53-MDM2 pathway [168,169]. In fact, in NB cells, it has been shown that N-MYC directly promotes MDM2 transcription, which in turn targets p53 for degradation [170]. More recently, it was shown that N-MYC could directly regulate p53 transcriptional activity in *MYCN*-amplified NB [171]. The authors showed that N-MYC bound to the C-terminal domain of p53, causing pronounced alterations of the expression of p53 target genes. This N-MYC-p53 interaction also led to the transcription of alternative p53 targets not induced at low N-MYC levels, further promoting the oncogenic effects of N-MYC overexpression [171]. That work discloses a new strategy of improving p53-mediated responses by targeting N-MYC, which sensitizes *MYCN*-amplified NB to chemotherapy.

By upregulating MDM2 levels, N-MYC might also indirectly inhibit TAp73 transcriptional activity [39] (Figure 1b). Some studies have suggested that overexpression of N-MYC in NB cells could decrease the expression levels of TAp73, by repressing its transcription [39]. Conversely, when TAp73 was overexpressed, the expression levels of N-MYC were reduced, therefore promoting neuronal differentiation, which pointed to an antagonistic role of these two transcription factors in NB proliferation and differentiation [36,172].

As a major therapeutic target for high-risk NB, several strategies have been proposed for inhibiting N-MYC function in NB (Figure 2). An example is the use of compounds 10058-F4 and 10074-G5, which block the N-MYC heterodimerization with MAX protein (that is involved in NB progression), leading to apoptosis in *MYCN*-amplified NB cells [173]. However, in vivo experiments with these compounds had limitations, namely the rapid metabolism of 10074-G5 to inactive metabolites resulted in tumor concentrations of 10074-G5 insufficient to inhibit c-MYC/MAX dimerization [174]. Additionally, 10058-F4 was rapidly metabolized, not reaching effective tumor concentrations, thus showing little or no efficacy against established xenografts [174]. Another strategy is based on the inhibition of AURKA, which is responsible for stabilizing N-MYC, protecting it from proteasomal degradation. In preclinical studies, inhibition of AURKA by MLN8237 (alisertib) efficiently induced N-MYC degradation, G2/M cell cycle arrest, apoptosis, and reduction in phosphorylation of the Aurora kinase substrate histone H3 in NB cells, and induced tumor regression in NB xenografts mice (Table 1) [10,175]. Additionally, some clinical trials are ongoing to investigate the combination of AURKA inhibitors with chemotherapeutic agents, namely irinotecan and temozolomide (Table 1) [176]. Another example is the inhibition of the bromodomain and extra-terminal domain (BET) family proteins, repressing N-MYC levels and transcriptional activity. Indeed, the inhibition of BET by compounds such as JQ1, OTX015 and GSK1324726A (I-BET726) is an additional strategy to inhibit *MYCN* expression in NB cells [2]. Clinical trials are underway with these compounds in several tumors, but not in NB yet [175,177]. It has also been demonstrated that abnormal activation of the PI3K/AKT/mTOR pathway was associated with *MYCN* amplification in high-risk NB [178]. Thus, research has been focused on finding inhibitors of PI3K, AKT and mTOR. Several of these agents have been developed, such as SF1126 (inhibitor of PI3K and mTOR, under clinical trials in NB (Table 1)) and MK2206 (inhibitor of AKT) [179]. The effect of these compounds in combination with chemotherapy are under investigation [10,177].

MDM2 can also stabilize *MYCN* mRNA and its translation, forming a positive feedback loop that promotes *MYCN* amplification, leading to growth and survival of NB cells [168]. This loop is independent of p53 [168]. Consistently, preclinical studies have evidenced that NB cells are very sensitive to inhibitors of MDM2, such as nutlin-3a, including in p53-null NB cells with *MYCN* amplification, which demonstrates a p53-independent mechanism of action of these drugs, namely by targeting the *MYCN* pathway [53,54,55] (Figure 2).

## 4. Crosstalk between miRNAs and p53/TAp73

miRNAs are a class of small single-stranded non-coding RNAs, having 20–22 nucleotides [180,181]. They are encoded within the introns or exons of protein-coding genes (30%) or in intergenic areas (70%) [182,183]. Over 30–60% of human genes are regulated by miRNAs [182]. miRNAs play important roles in regulating gene expression at the post-transcriptional level by targeting the 5′ untranslated region (UTR), coding regions or 3′UTR of mRNA. By binding to complementary regions of the mRNA through base pairing, they are able to inhibit translation or cause degradation of the mRNA [180,184].

Several studies have shown that miRNAs are crucial regulators of a wide variety of biological processes, including cell proliferation, differentiation, apoptosis, stress response, adhesion, migration, and invasion. In fact, they are commonly dysregulated in cancer [182,185], namely due to epigenetic mechanisms, suppressed expression by transcription factors, and mutations in miRNA biogenesis pathways [186,187]. miRNAs can act as oncogenes (“oncomiRs”) or tumor suppressor (“oncosuppressor miRs” or “TSmiRs”), depending on the functions of the target proteins they regulate. OncomiRs are overexpressed in malignant tumors, stimulating cell proliferation and inhibiting tumor suppressor genes, including p53 and TAp73 [188,189]. On the other hand, TSmiRs are downregulated in malignant tumors, repressing the development of neoplasms by inhibiting oncogenes. Individual miRNAs may also have dual functions, acting either as oncogenes or tumor suppressors [182,183,186].

Several studies have been conducted to better understand the role of miRNAs in the pathogenesis of NB. It has been shown that some miRNAs affect essential processes, such as apoptosis and differentiation [190]. Moreover, some miRNAs have already been described for their roles in the progression and inhibition of NB (Table 2). The use of miRNA-based drug targeting, to induce TSmiRs expression or oncomiRs inhibition, could be a promising approach for NB treatment, particularly to avoid the severe side effects from chemotherapy in pediatric cancers [190,191]. In addition, miRNAs have several advantages, including small size and stability in various adverse conditions (e.g., temperature or pH changes), they are easily quantifiable and commonly found in various body fluids (e.g., blood, urine, saliva, and plasma). Moreover, they have distinct modes of circulation (e.g., exosomes and microvesicles, protein complexes, high-density lipoproteins, apoptotic bodies) [182,183]. Interestingly, the potential of miRNA-based drug targeting has been suggested by Tivnan et al. [192]. In that study, nanoparticles conjugated with GD2 antibody and the tumor suppressor miR-34a caused a considerable reduction of NB growth [192]. Nevertheless, the use of miRNAs is still in the preclinical phase in NB. Further studies are needed to better understand the biological function of miRNAs in NB, as well as the delivery approach of miRNA-mediated therapy to improve its safety and validate its use in children [190,191].

**Figure 3:**
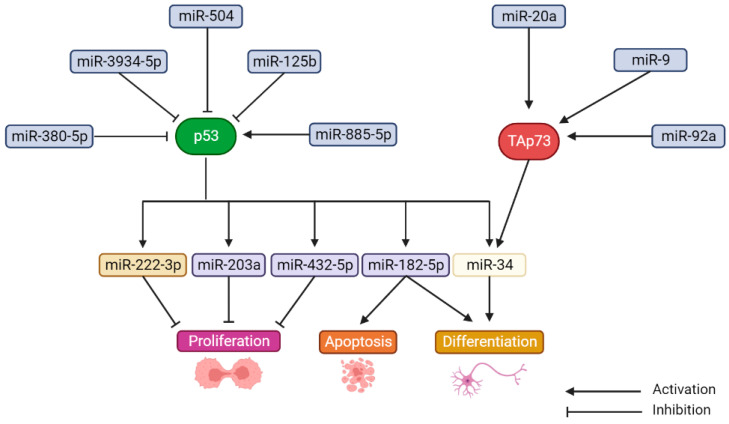
Transcriptional regulation of miRNAs by p53 and TAp73 in NB. Based on [204,213,214,215,216,217,218].

Some examples of miRNAs transcriptionally regulated by p53 and TAp73, and their biological outcomes in NB, are described in Figure 3 [188,193,194]. Recent works have also shown how mutp53 exhibits oncogenic properties by dysregulating the levels of specific miRNAs involved in epithelial–mesenchymal transition (EMT), therapeutic resistance, survival, among other cellular events (reviewed in [195]). In particular, mutp53 promotes the stability of miR-130b, miR-155 and miR-205, which are involved in invasion and metastasis [196]. Since mutp53 occurs more frequently in relapsed drug resistant NB, investigating the role of these emerging miRNAs, specifically in NB, might provide important insights into their utility as future therapeutic targets in high-risk or refractory tumors.

**Table 2:** Up- and downregulation of OncomiRs and TSmiRs, respectively, in NB.

miRNA	Target	Function	References
**Upregulated OncomiRs**			
miR-15a	*RECK*	Induces migration and invasion	[197]
miR-21	*PTEN, PDCD4,* *FOXO3A*	Induces proliferation and invasion	[198]
miR-23a	*CDH1*	Induces migration and invasion	[199]
miR-221	*NLK*	Induces proliferation and cell cycle progression	[200]
miR-380-5p	*TP53*	Increases proliferation and self-renewal	[201]
miR-558	*HPSE*	Induces proliferation, invasion, metastasis, and angiogenesis	[202]
miR-1303	*GSK3β,* *SFRP1*	Induces proliferation	[203]
miR-3934-5p	*TP53INP1*	Inhibits apoptosis and promotes viability	[204]
**Downregulated TSmiRs**			
Let-7	*MYCN*	Induces differentiation	[161,165]
miR-9	*MMP-14, TP73*	Inhibits invasion, metastasis, and angiogenesis	[205]
miR-15a/b	*MYCN*	Reduces proliferation, migration, and invasion	[206]
miR-16	*MYCN*	Reduces proliferation, migration, and invasion	[206]
miR-34a	*MYCN, E2F3,* *BCL2*	Induces cell cycle arrest and apoptosis; reduces angiogenesis	[207,208]
miR-192	*DICER1*	Inhibits proliferation and migration	[193,209]
miR-203	*KHDRBS1*	Inhibits invasion, proliferation, and migration	[210]
miR-338-3p	*PREX2a*	Inhibits proliferation and survival; induces cell cycle arrest	[211]
miR-1247	*ZNF346*	Inhibits proliferation; induces cell-cycle arrest and cell death	[212]

## 5. Conclusions

NB is a heterogenous disease with varied outcomes, from spontaneous regression to refractory growth and relapse. Despite the advances in NB treatment, up to 5% of NB patients diagnosed with favorable low- and intermediate-risk tumors eventually die from progressive disease, while survival of high-risk NB patients has plateaued at only 60% [219]. Therefore, the development of more effective therapeutic alternatives is urgently needed to improve the clinical outcomes and overall survival rates of NB patients.

The disruption of the p53-MDM2 interaction using MDM2 inhibitors is a compelling approach for NB patients that display low rates of p53 mutations. Despite the effectiveness of several classes of p53-MDM2 interaction inhibitors against NB, the displayed toxicity, and the development of resistance have restricted their clinical use. Improved inhibitors should be achieved, with better selectivity, lower systemic toxicity, and minor propensity for the development of genetic mutations that underlie the development of resistance.

This review addresses the dysregulation of the tumor suppressor proteins p53 and TAp73 in NB by several negative interactors, such as mutp53, ΔNp73, Itch, and AURKA. Targeting these undesirable interconnexions may represent new encouraging therapeutic strategies against NB. A particular attention should be given to the interplay between p53/TAp73 and N-MYC, one of the hallmarks of NB oncogenesis, as these interactions have great impact on the NB traits, including maintenance of stemness, metabolic plasticity, self-renewal, and promotion of proliferation. In fact, compiling data are herein provided showing the interest of combinatory regimes of inhibitors of N-MYC and MDM2.

Recurrent epigenetic, genetic, and molecular rearrangements are responsible for the rapid progression of NB to therapeutic resistance. In this regard, miRNAs may represent promising targets to counteract these resistance mechanisms. In fact, the emerging understanding of their roles in NB pathogenesis has provided new perspectives for novel NB diagnosis, prognosis, and even miR-targeted therapies.

With the recent progress in deciphering NB heterogeneity and origins by modern approaches, including single-cell transcriptomics [4,5], there is an enormous expectation for the identification of additional drug candidates, much more effective and safer, to enter clinical trials in the near future. In this review, compelling data are provided supporting the inclusion of therapies targeting the p53/TAp73 pathway in the treatment of NB, and particularly its aggressive MYC-driven forms.

## Figures and Tables

**Table 1:** Examples of targeted therapies under clinical trials for NB treatment.

Clinical Approach	Drugs Tested	Study Phase	Clinical Trials.gov Identifier or Ref.
Treatment with a MAPK inhibitor for relapsed or high-risk NB with activation mutations	Selumetinib sulfate	Phase 2	NCT03213691
Treatment with a ALK inhibitor for NB with ALK mutations	Crizotinib	Phase 1	NCT01121588
Combination therapy of ALK inhibitor (crizotinib) with chemotherapeutics	Crizotinib + dexrazoxane hydrochloride + topotecan hydrochloride + cyclophosphamide + doxorubicin + vincristine sulfate	Phase 1	NCT01606878
Combination therapy of ALK inhibitor (lorlatinib) with/without other chemotherapeutics	Lorlatinib + cyclophosphamide + topotecan	Phase 1	NCT03107988
Combination therapy of ALK inhibitor (ceritinib) with CDK 4/6 inhibitor (ribociclib)	Ceritinib + ribociclib	Phase 1	NCT02780128
Therapy with PI3K/mTOR inhibitor in relapsed or high-risk NB with PI3K/mTOR mutations	Samotolisib	Phase 2	NCT03213678
Treatment of NB with PI3K/mTOR inhibitor	SF1126	Phase 1	NCT02337309
Combination therapy of mTOR inhibitor (rapamycin) with multi-kinase inhibitor (dasatinib) with other chemotherapeutics	Rapamycin + dasatinib + irinotecan + temozolomide	Phase 2	NCT01467986
Combination therapy of mTOR inhibitor (temsirolimus) with perifosine	Temsirolimus + perifosine	Phase 1	NCT01049841
Combination therapy of AURKA inhibitor (alisertib) with chemotherapeutic agents	Alisertib + irinotecan + temozolomide	Phase 1/2	NCT01601535
Combination therapy of AURKA inhibitor (LY3295668 Erbumine) with/without other chemotherapeutics	LY3295668 Erbumine + topotecan + cyclophosphamide	Phase 1	NCT04106219
Combination therapy of MDM2 inhibitor (idasanutlin) with/without other chemotherapeutics or venetoclax	Idasanutlin + chemotherapy (cyclophosphamide/topotecan/fludarabine/cytarabine) or venetoclax	Phase 1/2	NCT04029688
Combination therapy of HDAC inhibitor (vorinostat) with 13-cis-retinoic acid (isotretinoin)	Vorinostat + isotretinoin	Phase 1	NCT01208454
Combination therapy of HDAC inhibitor (vorinostat) with bortezomib	Vorinostat + bortezomib	Phase 1	NCT01132911
Combination therapy of HDAC inhibitor (vorinostat) with ^131^I-MIBG in resistant or relapsed NB	Vorinostat + ^131^I-MIBG	Phase 1	NCT01019850

Source: https://clinicaltrials.gov/ (accessed on 30 October 2022).
